# Heteroscedastic sparse Gaussian process regression-based stochastic material model for plastic structural analysis

**DOI:** 10.1038/s41598-022-06870-9

**Published:** 2022-02-22

**Authors:** Baixi Chen, Luming Shen, Hao Zhang

**Affiliations:** School of Civil Engineering, The University of Sydney, Sydney, NSW 2006 Australia

**Keywords:** Civil engineering, Mechanical engineering

## Abstract

Describing the material flow stress and the associated uncertainty is essential for the plastic stochastic structural analysis. In this context, a data-driven approach-heteroscedastic sparse Gaussian process regression (HSGPR) with enhanced efficiency is introduced to model the material flow stress. Different from other machine learning approaches, e.g. artificial neural network (ANN), which only estimate the deterministic flow stress, the HSGPR model can capture the flow stress and its uncertainty simultaneously from the dataset. For validating the proposed model, the experimental data of the Al 6061 alloy is used here. Without setting a priori assumption on the mathematical expression, the proposed HSGPR-based flow stress model can produce a better prediction of the experimental stress data than the ANN model, the conventional GPR model, and Johnson Cook model at elevated temperatures. After the HSGPR-based flow stress model is implemented into finite element analysis, two numerical examples with synthetic material properties are performed to demonstrate the model’s capability in stochastic plastic structural analysis. The results have shown that with sufficient data, the distribution of the structural load carrying capacity at elevated temperatures and the variation of load–displacement curves during the loading and unloading processes can be accurately predicted by the HSGPR-based flow stress model.

## Introduction

The flow stress behavior takes an important role in plastic structural analysis. Many constitutive models have been proposed to describe the flow stress of material and they can mainly be divided into two types, i.e., phenomenological constitutive models and physics-based constitutive models. Phenomenological constitutive models include the Johnson Cook model^1,2^, Hockett-Sherby model^3^ and Arrhenius model^4^, etc. For physical-based constitutive modelling, there are Zerilli–Armstrong model^5^ and Rusinek–Klepaczko model^6^ etc. The above-mentioned conventional flow stress models could achieve good description in many situations, but the applications are limited by their mathematical expression. When there is a new alloy or a complex non-linear flow behavior, the original expression will become unreliable, and a new mathematical expression often needs to be developed. For example, Samantaray et.al^7^ modified the expression of the Zerilli–Armstrong model to make it suitable for the modified 9Cr-1Mo steel. However, the development of a new mathematical expression is non-trivial and can be time-consuming^8^.

To overcome the abovementioned limitations of the conventional constitutive approaches, machine learning has been introduced to model the flow stress behavior by many researchers^8–14^. Quan et al.^8^ used one of the popular machine learning algorithms, the artificial neural network (ANN), to evaluate the flow stress of Al7050 aluminum alloy and the obtained ANN-based flow stress model showed higher accuracy than the Arrhenius model. Besides the ANN model, the support vector regression was also used by Tang et al. to predict the flow stress of Al2519 aluminum alloy^15^. The high agreement between the experimental data and the machine learning-based flow stress model was observed in the above-mentioned studies. Unlike the phenomenological constitutive models and physical-based constitutive models, machine learning-based flow stress models do not require any pre-postulated mathematical model and can predict the flow behavior more efficiently and more conveniently, as long as sufficient amount of experimental data are available^8^.

Although the machine learning based flow stress models showed good performance in the previous studies, most of the existing machine learning based models are deterministic and do not consider the material uncertainty. Due to the manufacture error of the material and the variation of the chemical compositions, even the same material will show different behavior under the same conditions. For example, Aakash et.al^16^ reported that the flow stress-plastic strain curve of Al6061 aluminum alloys had large variation even under the same temperature. Without considering the material uncertainty, the reliability of the constitutive model cannot be guaranteed, and the simulation given by this constitutive model can be unreliable.

By keeping the advantage of machine learning and taking the material uncertainty into account, a Bayesian based machine learning algorithm, Gaussian process regression (GPR), has been adopted to model material behavior^17^. Different from other machine learning methods only providing the deterministic estimation, the GPR model can capture both the underlying relation and corresponding uncertainty of the data simultaneously via the Bayesian approach^18^. In recent years, GPR model has achieved wide applications in several areas, including structural reliability analysis^19^, material strength estimation^20^, and deformation forecasting^21^. However, the time complexity of the conventional GPR model is the cube of the data points ($O\left({n}^{3}\right)$). When more factors are included and the dataset become large, it will be hard to train and use the conventional GPR model. Hence, in order to improve the model efficiency, an enhanced GPR model, i.e. heteroscedastic sparse Gaussian process regression (HSGPR)^22^, is introduced to capture the flow stress behavior in the present work. Compared with the conventional GPR model, the HSGPR model uses the sparse technology to achieve higher efficiency and reduce the time complexity from $O\left({n}^{3}\right)$ to $O\left(nmd+n{m}^{2}\right)$^22,23^, where *n* is the number of samples, *d* is the dimension of the input and *m* is the number of the basis functions with *d* <  < *n* and *m* <  < *n*. Besides, different from the conventional GPR model, which usually uses the constant uncertainty model, the HSGPR model encodes a more advanced input-dependent uncertainty model in its structure. The advanced uncertainty model enables the HSGPR model to produce more accurate uncertainty estimation than the conventional GPR model^22^.

In this paper, the HSGPR model which takes the temperature and plastic strain as input variable and flow stress as output variable is used to identify the stochastic temperature-dependent elasto-plastic behavior. Without loss of generality, the experimental data of the Al6061 aluminum alloy at elevated temperatures are used in the present work to verify the HSGPR-based flow stress model. The obtained HSGPR-based flow stress model is then compared with the Johnson Cook model, which was often used to describe the Al6061 aluminum alloy in the previous studies^24–27^, ANN model and the conventional GPR model. For the purpose of completeness, the methodology of numerically implementing the HSGPR model-based flow stress model into finite element analysis and stochastic plastic structural analysis is introduced in this work as well. Finally, two numerical examples with the synthetic flow stress data are used to demonstrate the capability of the HSGPR-based flow stress model in stochastic plastic structural analysis.

## HSGPR-based flow stress model

### Basic theory of HSGPR

To achieve the sparse Gaussian process regression, the basis functions approach is used here^22,23^. As shown in the equations below, the output $y$ is regarded as a linear combination of $m$ nonlinear basis functions $\phi \left( {\varvec{x}} \right)$ of the inputs ${\varvec{x}}$:

1 $$ y = \phi \left( {\varvec{x}} \right){\varvec{w}} + \in $$

2 $$ \phi \left( {\varvec{x}} \right) = \left[ {\phi_{1} \left( {\varvec{x}} \right), \ldots ,\phi_{j} \left( {\varvec{x}} \right), \ldots , \phi_{m} \left( {\varvec{x}} \right)} \right] $$

3 $$ \phi_{j} \left( {\varvec{x}} \right) = {\text{exp}}\left[ { - \frac{1}{2}\left( {{\varvec{x}} - {\varvec{p}}_{{\varvec{j}}} } \right)^{T} {{\varvec{\Gamma}}}_{{\varvec{j}}}^{T} {{\varvec{\Gamma}}}_{{\varvec{j}}} \left( {{\varvec{x}} - {\varvec{p}}_{{\varvec{j}}} } \right)} \right] $$

where ${\varvec{x}} \in {\mathbb{R}}^{1 \times d}$ is the input vector and $d$ is the dimension of the input, $y \in {\mathbb{R}}^{1}$ is the output, $\phi_{j} \left( {\varvec{x}} \right)$ is the basis function. In this paper, the radial basis function kernel is chosen as the basis function for the HSGPR model and its expression is presented in Eq. (3). $m$ is the number of the basis functions,$\user2{ w}$ is the weight of basis functions and has the prior distribution ${\mathcal{N}}\left( {0,{\varvec{A}}} \right)$ where ${\varvec{A}}$ is an $m \times m$ constant diagonal matrix and represents the covariance matrix of the prior distribution, ${\varvec{p}}_{{\varvec{j}}} \in {\mathbb{R}}^{1 \times d}$ and ${{\varvec{\Gamma}}}_{{\varvec{j}}} \in {\mathbb{R}}^{d \times d}$ are the parameters of the basis function and ${{\varvec{\Gamma}}}_{{\varvec{j}}}$ is a diagonal matrix. $\in$ is the uncertainty of the output and is assumed to follow the normal distribution ${\mathcal{N}}\left( {0,\sqrt \beta^{2} } \right)$ and $\beta$ reflects the intensity of the uncertainty. To consider the input dependent uncertainty and enhancing the uncertainty estimation, the HSGPR model regards $\beta$ as the linear combination of the nonlinear basis functions as given below.

4 $$ \beta \left( {\varvec{x}} \right) = {\text{exp}}\left( {\phi \left( {\varvec{x}} \right){\varvec{u}} + b} \right) $$

where $\phi \left( {\varvec{x}} \right)$ is same as the basis functions in Eq. (2), ${\varvec{u}}$ is the weight of the basis functions and has the prior distribution ${\mathcal{N}}\left( {0,{\varvec{N}}} \right)$ where ${\varvec{N}}$ is an $m \times m$ diagonal matrix, and *b* is the constant part of Eq. (4) and is used to consider the situation of constant uncertainty intensity. The exponential function used here is to make $\beta$ positive.

In terms of training dataset $\left( {{\varvec{X}}^{\user2{*}} ,{\varvec{y}}^{\user2{*}} } \right)$, ${\varvec{X}}^{\user2{*}} = \left\{ {{\varvec{x}}_{{\varvec{i}}}^{\user2{*}} } \right\}_{i = 1}^{n} \in {\mathbb{R}}^{n \times d}$ is the $n \times d$ matrix storing $n$ training inputs vectors and ${\varvec{y}}^{\user2{*}} = \left\{ {y_{i}^{*} } \right\}_{i = 1}^{n} \in {\mathbb{R}}^{n \times 1}$ is the vector storing $n$ training outputs. The log marginal likelihood ${\text{ln}}p\left( {{\varvec{y}}^{\user2{*}} } \right)$ of the training data can be expressed as

5 $$ \begin{aligned} \ln p\left( {{\varvec{y}}^{\user2{*}} } \right) = & - \frac{1}{2}\left( {{{\varvec{\Phi}}}\overline{\user2{w}} - {\varvec{y}}^{\user2{*}} } \right)^{T} {\varvec{B}}\left( {{{\varvec{\Phi}}}\overline{\user2{w}} - {\varvec{y}}^{\user2{*}} } \right) \\ & \quad + \frac{1}{2}\ln \left| {\varvec{B}} \right| - \frac{n}{2}\ln 2{\uppi } - \frac{1}{2}\overline{\user2{w}}^{T} \user2{A\overline{w}} + \frac{1}{2}\ln \left| {\varvec{A}} \right| \\ & \quad - \frac{1}{2}\ln \left| {{{\varvec{\Phi}}}^{{\varvec{T}}} {\varvec{B}}{{\varvec{\Phi}}} + {\varvec{A}}} \right| - \frac{1}{2}{\varvec{u}}^{T} {\varvec{Nu}} + + \frac{1}{2}\ln \left| {\varvec{N}} \right| - \frac{m}{2}\ln 2{\uppi } \\ \end{aligned} $$

where ${{\varvec{\Phi}}} = \left\{ {\phi \left( {{\varvec{x}}_{{\varvec{i}}}^{\user2{*}} } \right)} \right\}_{i = 1}^{n} \in {\mathbb{R}}^{n \times m}$ is the $n \times m$ matrix storing the basis functions for each training inputs, ${\varvec{B}}$ is the $n \times n$ diagonal matrix with diagonal elements ${\varvec{B}}\left( {i.i} \right) = \beta \left( {{\varvec{x}}_{{\varvec{i}}}^{\user2{*}} } \right)$ and represents the uncertainty intensity at the training inputs ${\varvec{X}}^{\user2{*}}$, and $\overline{\user2{w}} = \left( {{{\varvec{\Phi}}}^{{\varvec{T}}} {\varvec{B}}{{\varvec{\Phi}}} + {\varvec{A}}} \right)^{ - 1} {{\varvec{\Phi}}}^{{\varvec{T}}} {\varvec{By}}^{\user2{*}}$**.**

The parameters mentioned above can be determined by the training process. The training process is conducted by maximizing the log marginal likelihood of the training data. The optimization is achieved by using the quasi-Newton approach. After training the model, the parameters and weights in the basis functions can be updated to the optimal values. The output $y$ at the unseen input ${\varvec{x}}$ is normally distributed as ${\mathcal{N}}\left( {\mu ,\delta^{2} } \right)$ with

6 $$ \mu = \overline{\phi }\left( {\varvec{x}} \right)\overline{\user2{w}} $$

7 $$ \delta^{2} = v + \beta $$

8 $$ v = \overline{\phi }\left( {\varvec{x}} \right) {{\varvec{\Sigma}}}^{ - 1} \overline{\phi }\left( {\varvec{x}} \right)^{T} $$

9 $$ \beta = {\text{exp}}\left( {\overline{\phi }\left( {\varvec{x}} \right)\overline{\user2{u}} + \overline{b}} \right) $$

where $\overline{\phi }\left( {\varvec{x}} \right)$ is the updated basis functions; $\overline{\user2{w}}$, $\overline{\user2{u}}$ and $\overline{b}$ are the updated parameters and ${{\varvec{\Sigma}}} = {{\varvec{\Phi}}}^{{\varvec{T}}} {\varvec{B}}{{\varvec{\Phi}}} + {\varvec{A}}$ is a $m \times m$ matrix. $\mu$ and $\delta$ are the expectation and standard deviation of the prediction of the unseen input ${\varvec{x}}$.

### HSGPR-based flow stress model

**Figure 1:**
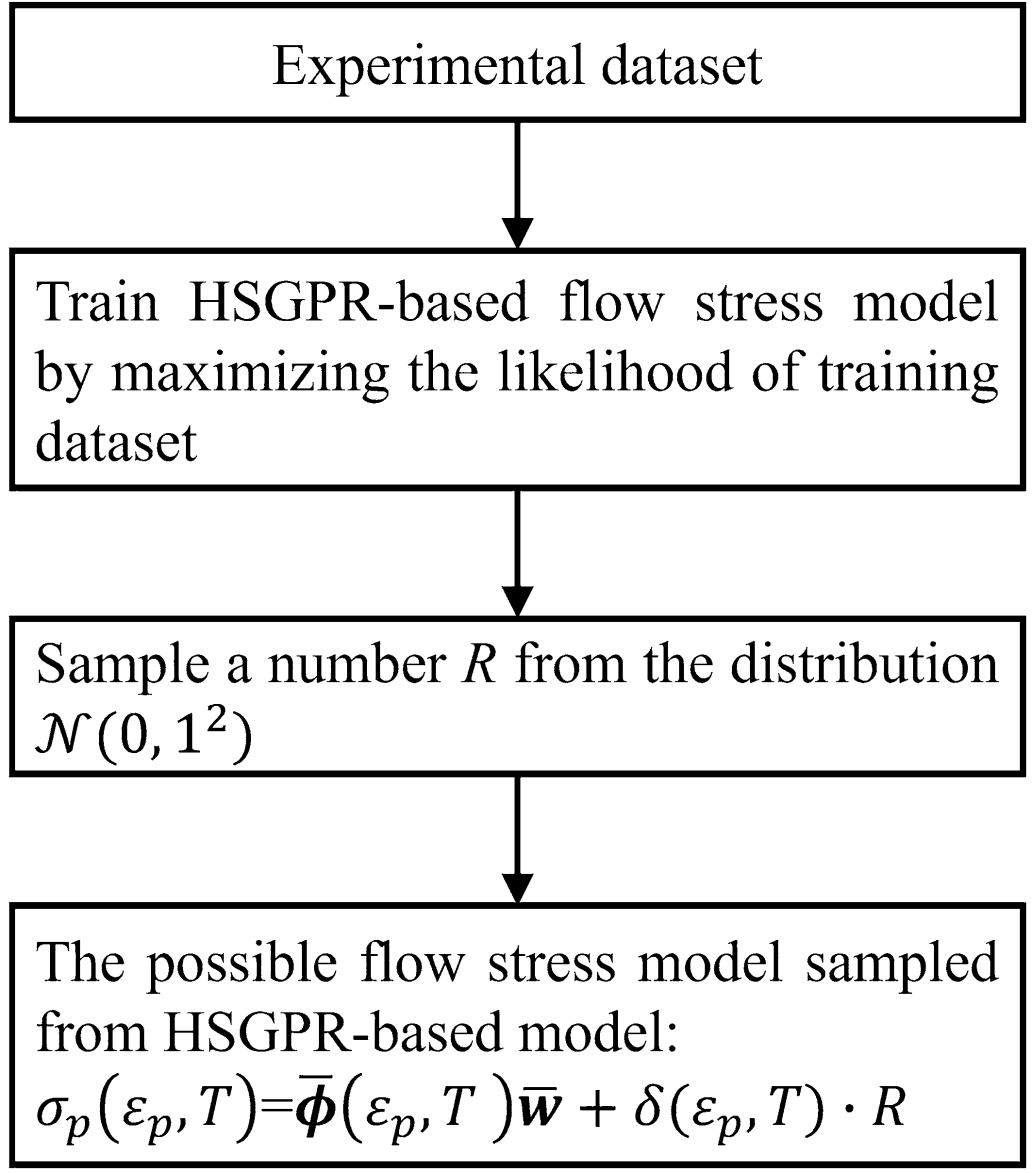
The flow chart of establishing HSGPR-based stochastic flow stress model.

When the HSGPR model is used to model the material flow stress $\sigma_{p}$ at elevated temperatures, the plastic strain $\varepsilon_{p}$ and the temperature $T$ (K) are used as the input variables and the flow stress $\sigma_{p}$ (MPa) is used as the output variable. The number of basis functions can be determined by trial and error. A larger number of basis functions can give higher accuracy but will increase the model complexity. Based on Eq. (6) to Eq. (9) and the Gaussian prior distribution assumed before, the obtained HSGPR-based stochastic flow stress model $\sigma_{p} \left( {\varepsilon_{p} , T} \right)$ follows the Gaussian distribution with the mean of $\overline{\phi }\left( {\varepsilon_{p} ,T } \right)\overline{\user2{w}}$ and the variance of $\delta^{2} \left( {\varepsilon_{p} ,T} \right)$, as expressed below

10 $$ \sigma_{p} \left( {\varepsilon_{p} , T} \right) = \overline{\phi }\left( {\varepsilon_{p} ,T } \right)\overline{\user2{w}} + \delta \left( {\varepsilon_{p} ,T} \right) \cdot R $$

11 $$ \delta^{2} \left( {\varepsilon_{p} ,T} \right) = \overline{\phi }\left( {\varepsilon_{p} ,T} \right) {{\varvec{\Sigma}}} \overline{\phi }\left( {\varepsilon_{p} ,T} \right)^{T} + {\text{exp}}\left( {\overline{\phi }\left( {\varepsilon_{p} ,T} \right)\overline{\user2{u}} + \overline{b}} \right) $$

where $R$ is a standard normal distribution of ${\mathcal{N}}\left( {0,1^{2} } \right)$. The possible curve of the HSGPR based stochastic flow stress model can be sampled by sampling standard normal random variable $R$ first and then substituting the sampled random variable into Eq. (10). The procedure of the HSGPR-based flow stress modelling is illustrated as a flow chart in Fig. 1.

### Model evaluation

To evaluate the accuracy of the obtained flow stress models, the average absolute relative error (AARE) is used here. The model with lower AARE has higher accuracy.

The expression of AARE is presented below:

12 $$ AARE = \frac{1}{N}\mathop \sum \limits_{i = 1}^{N} \frac{{\left| {\sigma_{pi}^{exp} - \sigma_{pi}^{pre} } \right|}}{{\sigma_{pi}^{exp} }} $$

where $\sigma_{pi}^{exp}$ and $\sigma_{pi}^{pre}$ are the *i*th experimental flow stress and the *i*th predicted mean flow stress of the model respectively, and $N$ is the number of the data.

To evaluate the uncertainty of the flow stress predicted by the models, the mean log likelihood (MLL) of the experimental data is introduced here. The MLL reflects the average likelihood of obtaining the experimental data under the given model. The model with a higher MLL means that it is more likely to reproduce the data under the given model and the material uncertainty predicted by this model has higher accuracy.

The expression of MLL is given below:

13 $$ MLL = \frac{1}{N}\mathop \sum \limits_{i = 1}^{N} \left( { - \frac{{\left( {\sigma_{pi}^{exp} - \sigma_{pi}^{pre} } \right)^{2} }}{{2\delta_{i}^{2} }} - \frac{1}{2}\ln \left( {\delta_{i}^{2} } \right)} \right) - \frac{1}{2}\ln \left( {2\pi } \right) $$

where $\delta_{i}$ is the standard deviation of the *i*th flow stress predicted by the model.

## Application for material modelling

### Material data

**Figure 2:**
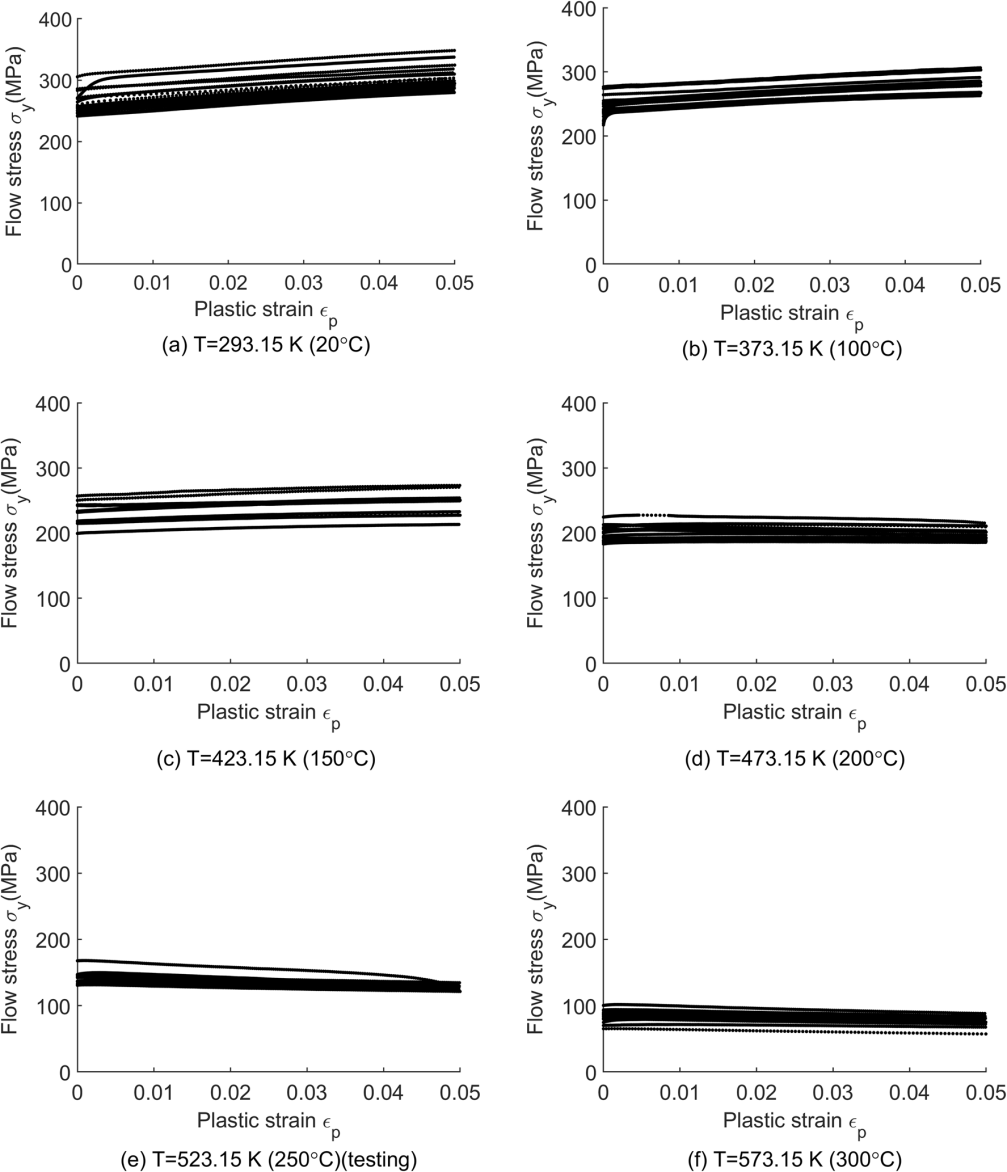
The experimental flow stress-plastic strain data of Al6061 aluminum alloys at the temperature of (**a**) 20 °C, (**b**) 100 °C, (**c**) 150 °C, (**d**) 200 °C, (**e**) 250 °C and (**f**) 300°C^28^.

Without loss of generality, the experimental flow stress-plastic strain data of Al6061 aluminum alloy from the literature^28^ are applied in the present work. There were six temperatures (20, 100, 150, 200, 250, 300 °C) considered in the experiments. The experiment data obtained by the uniaxial tension test conducted on a standard “dog bone” type coupon by MTS 810 (10-kip capacity). Design of the uniaxial tension test followed the guidelines in ASTM E8. A total of 98 stress–strain curves (totally 24,011 data points) are used here. The detailed breakdown of the obtained curves is illustrated in Table 1 and all data curves are presented in Fig. 2.

**Table 1:** Number of the stress–strain curves for each lot and each temperature.

	Temperature (°C)					
	20	100	150	200	250	300
Number of curves	19	18	12	18	12	19
Number of data points	4513	5194	3910	4168	2488	3738

Kolmogorov–Smirnov hypothesis test is conducted on the flow stress data at different temperature and different plastic strain to examine if the flow stress follows the Gaussian distribution. The p-values of the hypothesis test on the flow stress are presented in Table 2. As demonstrated in Table 2, the test does not reject the hypothesis, indicating that the flow stress obeys the Gaussian distribution, at the 5% significance level. Hence, it is reasonable to use the normal distribution for the uncertainty $\in$ in the HSGPR model and Eq. (10) to describe the stochastic behavior of the flow stress.

**Table 2:** The *P*-value of the Kolmogorov–Smirnov hypothesis test on the flow stress data at various temperatures and plastic strains.

*P*-value		Temperature (°C)					
		20	100	150	200	250	300
Plastic strain $\varepsilon_{p}$	0.00	0.61	0.92	0.84	0.57	0.99	0.79
	0.01	0.51	0.52	0.95	0.78	0.94	0.85
	0.02	0.65	0.76	0.93	0.99	0.98	0.92
	0.03	0.62	0.89	0.94	0.98	0.98	0.95
	0.04	0.58	0.98	0.60	0.96	0.99	0.79
	0.05	0.54	0.98	0.52	0.62	0.96	0.71

In addition, the experimental data at temperature of 250 °C are used as the testing dataset for the HSGPR model, while the rest of data (21,523 data points) are used as the training dataset for the ANN model and HSGPR model and conventional GPR model.

## Results and discussion

Four models, namely, the Johnson Cook (JC) model^29^, the ANN model, the HSGPR model, and the conventional GPR model are used to model the flow stress of the Al6061 aluminum alloys at elevated temperatures.

The Johnson–Cook flow stress model was used by many researchers to model the flow behavior of Al6061 aluminum^24–27^. The formulation of JC model is expressed in Eq. (14).

14 $$ \sigma_{p} = \left[ {c_{1} + c_{2} \varepsilon_{p}^{{c_{3} }} } \right]\left[ {1 - \left( {\frac{{T - T_{r} }}{{T_{m} - T_{r} }}} \right)^{{c_{4} }} } \right] $$

where $\sigma_{p}$ (MPa) is the flow stress, $\varepsilon_{p}$ is the plastic strain, $T$ (K) is the temperature of material, $c_{1}$, $c_{2}$, $c_{3}$ and $c_{4}$ are the model parameters, $T_{r}$ is the room temperature of 293.15 K (i.e. 20 °C) and $T_{m}$ is the melt temperature of 925 K for the Al6061 aluminum alloy.

The parameters $c_{1}$, $c_{2}$, $c_{3}$ of JC model are calibrated by the stress–strain curves at room temperature (i.e. 20 °C) first. The Pearson correlation coefficients between $c_{1}$ and $c_{2}$, and between $c_{1}$ and $c_{3}$ are -0.002 and -0.021 respectively, which means that the correlation is negligible and $c_{1}$ is independent of $c_{2}$ and $c_{3}$^30^. As the Pearson correlation coefficients between $c_{2}$ and $c_{3}$ is 0.96, $c_{2}$ and $c_{3}$ are assumed to be linearly correlated with each other here. By conducting Kolmogorov–Smirnov test, the parameters $c_{1}$,$c_{2}$ and $c_{3}$ follow normal distribution ${ \mathcal{N}}\left( {260.72,17.32^{2} } \right) {\text{MPa}}$, ${\mathcal{N}}\left( {504.26,106.99^{2} } \right)$ MPa and ${\mathcal{N}}\left( {{ }0.82{ },0.14^{2} } \right)$ respectively at the 5% significance level. The parameter $c_{4}$ is calibrated by the data of zero plastic strain. $c_{4}$ obeys the normal distribution ${ \mathcal{N}}\left( {1.277,0.069^{2} } \right)$ at the 5% significance level.

**Figure 3:**
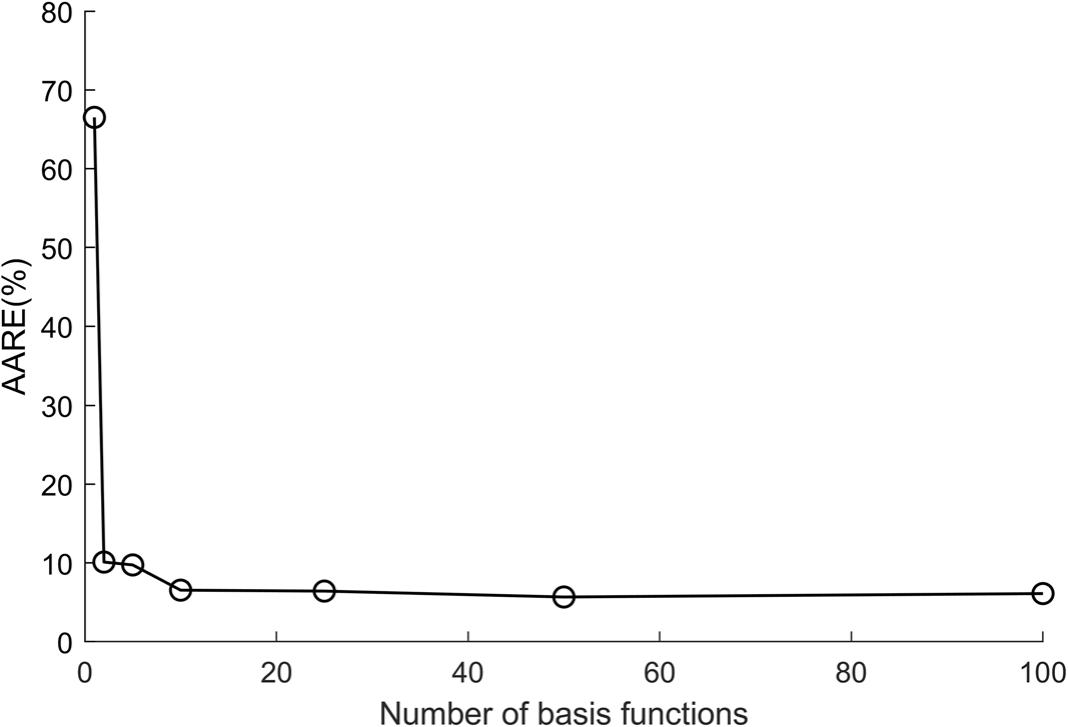
The relationship between the AARE on the testing dataset and the number of basis functions m.

The HSGPR-based flow stress model for the Al6061 aluminum alloys is established by the approach described in “Section HSGPR-based flow stress model”. In order to determine the suitable number of basis functions *m*, the relationship between the AARE on the testing dataset and *m* is drawn in Fig. 3. It can be observed from Fig. 3 that the improvement of the model accuracy is negligible when *m* is larger than 10. Hence, 10 basis functions are sufficient to produce a good estimate and more basis functions will only increase the model complexity without obvious contribution to the model accuracy. For the sake of the model efficiency and accuracy, *m* = 10 is adopted in the rest of the paper. With *m* = 10, the optimal parameters of the HSGPR model can be obtained by the training process and presented in “Appendix A” of Supplementary Information.

The conventional GPR model and the ANN model are established by using the MATLAB toolbox as well for comparison. For the GPR model, the squared exponential kernel function is used as the kernel function, and the subset of the data points approximation is set as the fitting method to address the efficiency issue. The quasi-Newton method is used to optimize the parameters in the GPR model and the default value in MATLAB is used for all other settings of the GPR model. The optimal parameters of the GPR model (i.e., the signal standard deviation, the characteristic length scales and the noise standard deviation) are 228.8175, 244.4820 and 17.8537 respectively. The ANN structure is adopted from literature^9^ directly. As demonstrated in the reference^9^, the network with one hidden layer consisting of 20 nodes is the suitable setting for estimating the flow stress.

Since the flow stress model needs to be called to predict the flow stress at each integration point and each increment step in the finite element analysis (FEA), the prediction time of the data-driven flow stress model will significantly influence the efficiency of the model in the FEA. The prediction time of the ANN model, HSGPR model and GPR model for 10,000 input points in the MATLAB platform are 0.019 s, 0.011 s and 26.78 s, respectively. Compared with the conventional GPR model, the HSGPR model and ANN model have lower time consumption during the prediction, which means they have higher efficiency in the FEA.

The AARE and MLL of the three machine learning models (namely, the HSGPR model, GPR model and ANN model) on the training and testing dataset are presented in Table 3. Since only deterministic prediction is given by the ANN model, only the AARE of the ANN model is calculated. The ANN model, HSGPR model and GPR model have similar AARE on the training stage, while the performance of ANN model on the testing dataset is the worst among the three models with the AARE of 12.4%. In addition, with the advanced uncertainty model, the HSGPR model presents obviously larger MLL and smaller AARE than the GPR model on the testing dataset. The larger MLL means the testing dataset is more likely to be reproduced by the HSGPR model than the GPR model.

**Table 3:** AARE and MLL of ANN model, HSGPR-based flow stress model and GPR-based flow stress model on the training and testing dataset.

Dataset	Model	AARE (%)	MLL
Training	HSGPR	5.2	− 4.04
	GPR	5.7	− 4.23
	ANN	5.2	–
Testing	HSGPR	6.5	− 3.74
	GPR	8.7	− 7.08
	ANN	12.4	–

In order to further evaluate the generalization ability of the three machine learning approaches, the cross-validation procedure is performed here as well. The data points at one selected temperature are excluded for validation, while the rest of the data are used for training. Hence, there are totally 6 cross-validation tests. The AARE of the three models on the excluded validation set is demonstrated in Table 4. In the validation test 1 and 6, the validation temperature is not covered by the training dataset, so the model extrapolation ability can be tested. Notably, the HSGPR model have superior accuracy over the GPR model and ANN model on extrapolation. Besides, the performance of the HSGPR model on the interpolation validation set (No. 2–5) is also better than the other two models. It can be concluded that the HSGPR model has better generalization ability than the conventional GPR model and ANN model on flow stress prediction.

**Table 4:** AARE of ANN-based flow stress model, HSGPR-based flow stress model, GPR-based flow stress model on cross validation set.

Validation No	Excluded temperature for validation (°C)	AARE (%)		
		HSGPR	GPR	ANN
1	20	9.9	30.0	17.2
2	100	8.6	8.7	8.5
3	150	4.2	4.4	7.2
4	200	4.5	6.6	4.5
5	250	6.5	8.7	12.4
6	300	8.1	33.1	23.6

As a conventional constitutive model, the JC model is directly evaluated using the whole dataset (including all the experimental data). For comparison, the three machine learning approaches are also evaluated on the whole dataset and the results are listed on Table 5. Due to the limitation of the mathematical expression, the performance of the JC model is worse than the machine learning approach. The HSGPR model has the smallest AARE and the largest MLL among all the models, which means that the HSGPR model shows better performance than the other models.

**Table 5:** AARE and MLL of the ANN model, HSGPR-based flow stress model, GPR-based flow stress model and JC model on the whole dataset.

Model	AARE (%)	MLL
HSGPR	5.3	− 4.01
GPR	6.0	− 4.21
ANN	6.0	–
JC	31.1	− 10.18

**Figure 4:**
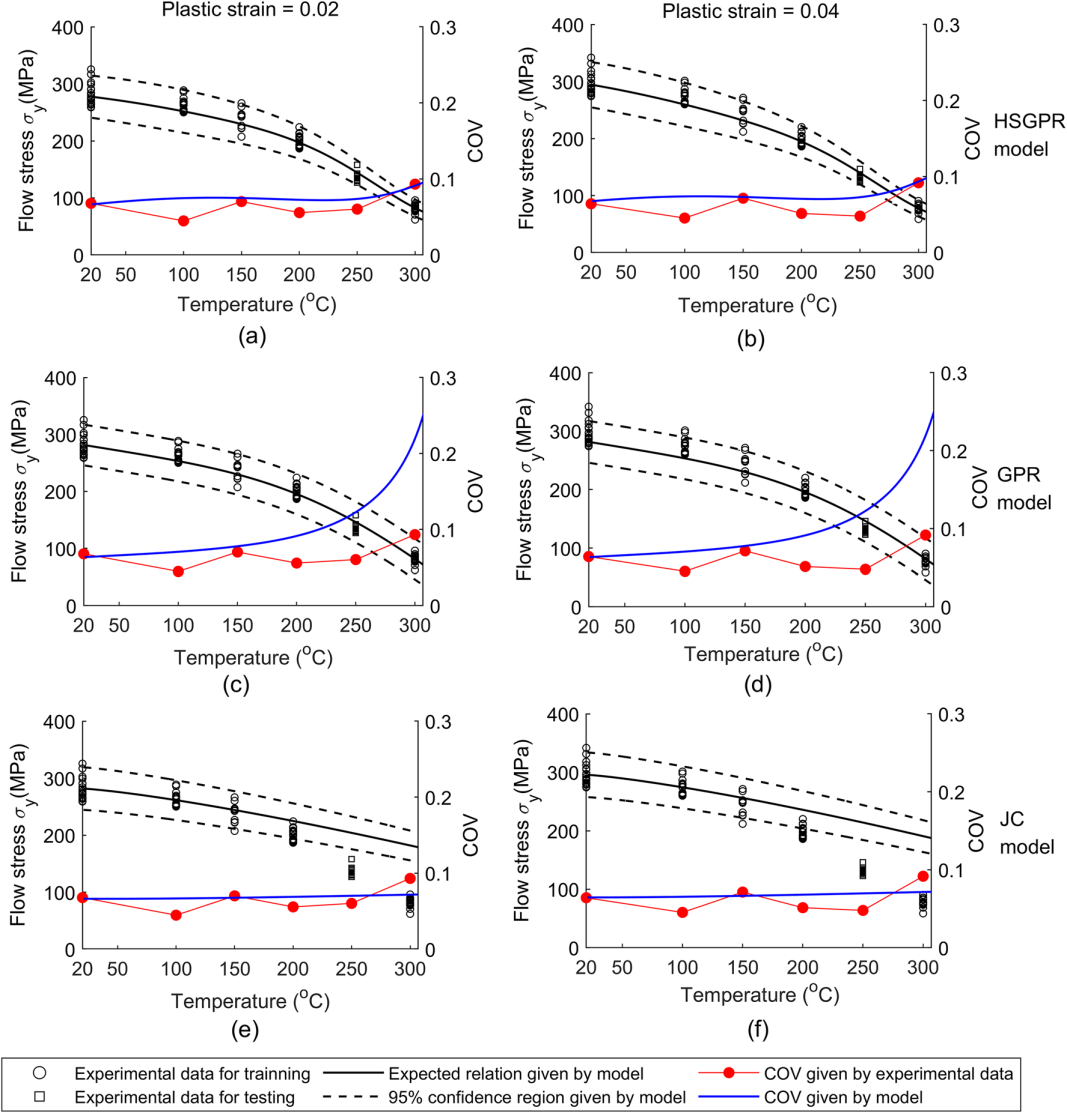
The obtained HSGPR-based flow stress model, GPR-based flow stress model and JC model with respect to the temperatures at the plastic strains of 0.02 and 0.04. (**a**) HSGPR model at the plastic strains of 0.02, (**b**) HSGPR model at the plastic strains of 0.04, (**c**) GPR model at the plastic strains of 0.02, (**d**) GPR model at the plastic strains of 0.04, (**e**) JC model at the plastic strains of 0.02, (**f**) JC model at the plastic strains of 0.04.

**Figure 5:**
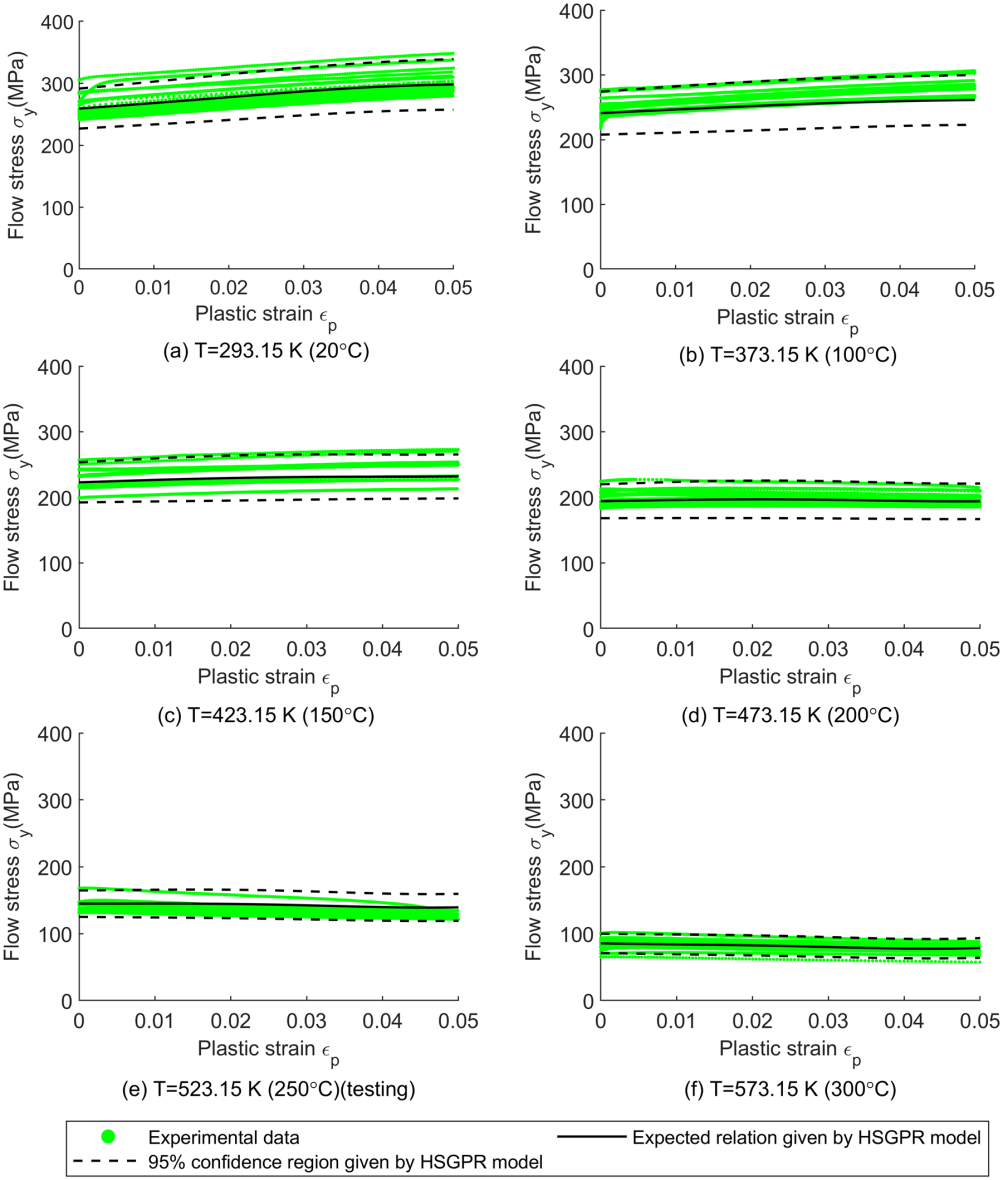
The flow stress obtained using the HSGPR-based model with respect to the plastic strain at the temperature of (**a**) 20 °C, (**b**) 100 °C, (**c**) 150 °C, (**d**) 200 °C, (**e**) 250 °C and (**f**) 300 °C.

**Figure 6:**
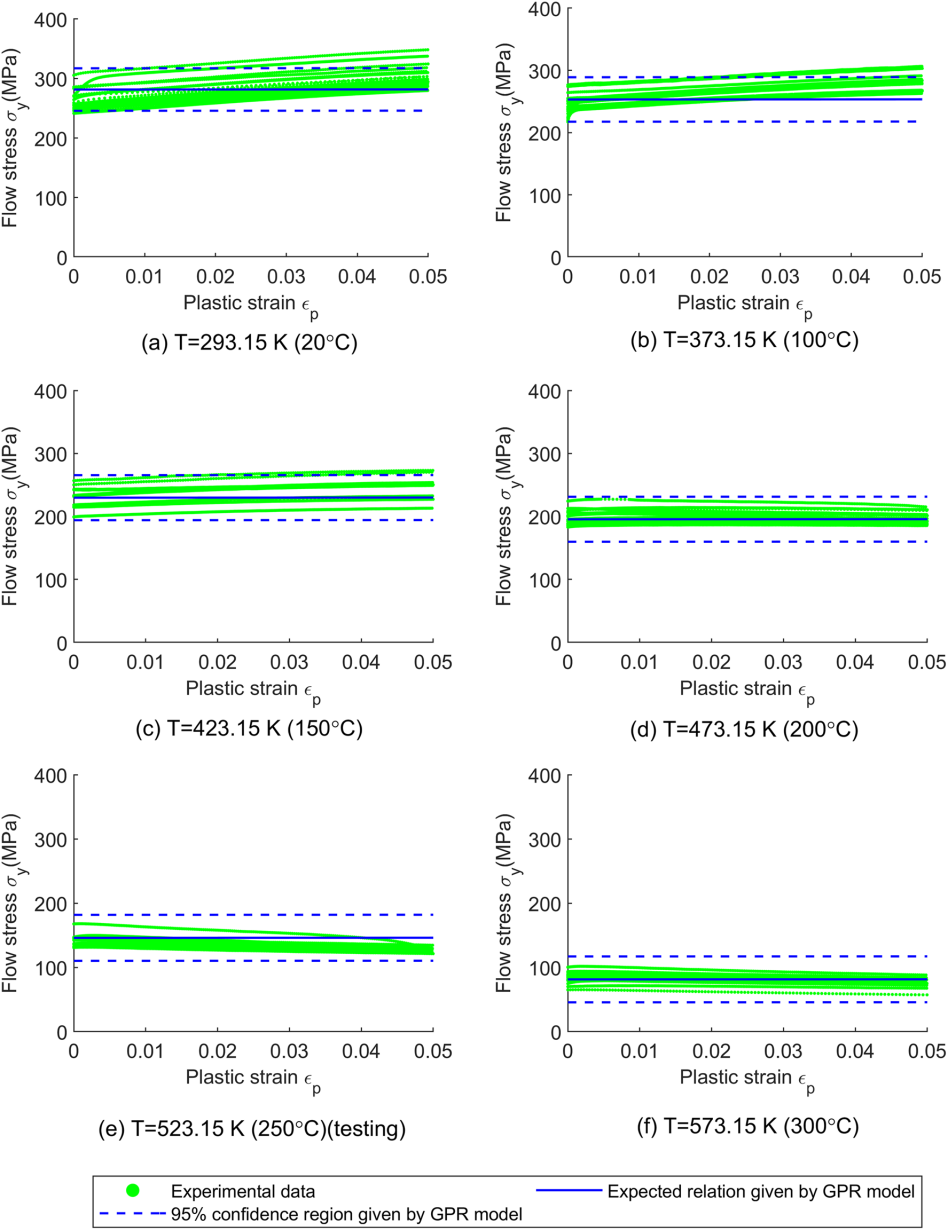
The flow stress obtained using GPR-based model with respect to the plastic strain at the temperature of (**a**) 20 °C, (**b**) 100 °C, (**c**) 150 °C, (**d**) 200 °C, (**e**) 250 °C and (**f**) 300 °C.

**Figure 7:**
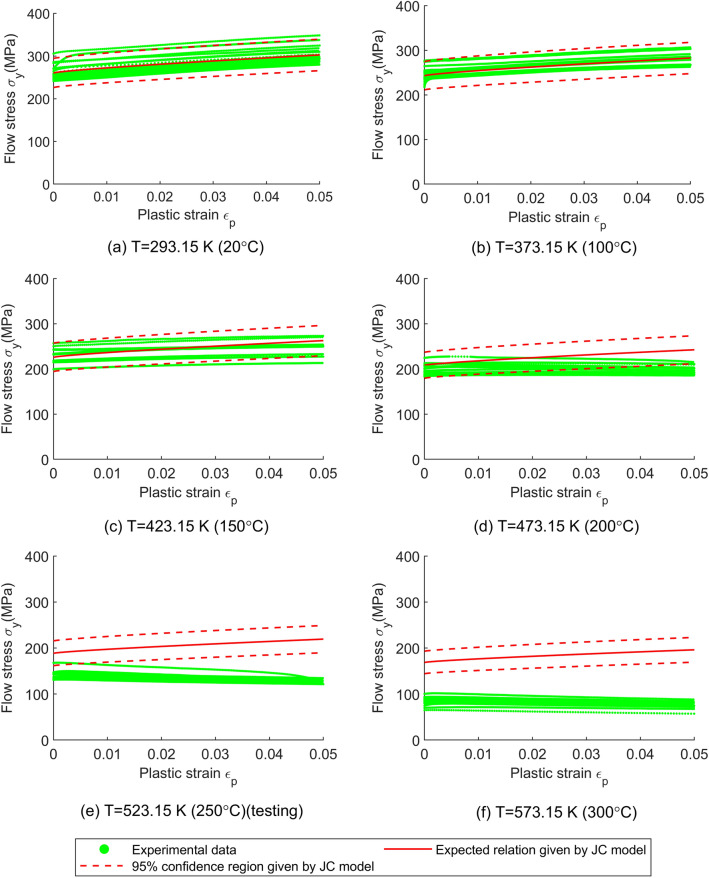
The flow stress obtained using the JC model with respect to the plastic strain at the temperature of (**a**) 20 °C, (**b**) 100 °C, (**c**) 150 °C, (**d**) 200 °C, (**e**) 250 °C and (**f**) 300 °C.

As for the material uncertainty predicted by the models, it can be visualized by the 95% confidence region (i.e. ${\text{mean}} \pm 2 \times {\text{standard deviation}}$) and the coefficient of variation (COV, i.e. ${\text{standard deviation}}/{\text{mean}}$) of the flow stress model and is plotted in Figs. 4, 5, 6, 7. Since the ANN cannot provide the material uncertainty, only the HSGPR-based flow stress model, GPR-based flow stress model and JC model are plotted and discussed in the next.

At the low temperatures (20 °C to 150 °C), it can be observed from Figs. 4, 5, 6, 7 that the 95% confidence region given by the three models could cover most of the data at low temperatures. Besides, as shown in Fig. 4, the COVs given by the three models are similar and are close to the COV given by the experimental data at the temperatures of 20 °C to 150 °C.

At the high temperatures (200 °C to 300 °C), the mean curves and material uncertainty estimated by the HSGPR model are consistent with the experimental data, while the material uncertainty is overestimated by the GPR model and the COV given by GPR model is much larger than the experimental value. It is notable to mention that the advanced uncertainty model within HSGPR model results in the better uncertainty estimation than the GPR model. In addition, as limited by its mathematic expression, the JC model cannot describe the severe temperature softening of Al6061 alloys at high temperatures. As shown in Fig. 7, the estimated flow stress given by the JC model deviates from the experiment data at high temperature, which causes the high AARE of the JC model in Table 5. The poor performance of the JC model for the Al6061 alloy at high temperatures is consistent with the results in the work of Fan et.al.^25^. Although it is possible to modify the JC model to improve the accuracy, those modifications are material-dependent and need to postulate the mathematical expression as well^31^. Establishing a new mathematic expression of the modified JC model for each new material is always a time-consuming work and sometimes a suitable modification is hard to find. Different from the JC model, the HSGPR-based flow stress model is a material-independent data-driven flow stress model. Without postulating the specific mathematical form, the HSGPR framework can be used to establish a flow stress model directly from the material data with high accuracy.

In addition, it should be admitted that the current HSGPR based model use the uncertainty model $\in$ with the normal distribution to approximate the uncertainty of material data. If the data deviated from the normal distribution, the uncertainty $\in$ with other distribution needs to be used. The new distribution of $\in$ can be selected by trial and error, and the distribution, which can produce the maximum MLL, is regarded as the suitable one.

## Application in plastic stochastic structural analysis

Since the HSGPR-based stochastic flow stress model is introduced for the first time in the current work and has not been implemented in the finite element analysis (FEA) for plastic stochastic structural analysis previously, the numerical implementation of the HSGPR-based flow stress model into the FEA for plastic stochastic structural analysis is proposed in this section. And the model is then validated by two numerical examples with the synthetic material data.

### Numerical implementation in FEA

The obtained HSGPR-based stochastic flow stress model can be combined with the von Mises yield criteria to describe the plastic behavior of the material in the FEA. The yield function with the HSGPR-based flow stress model can be expressed as

15 $$ f\left( {\overline{\sigma },\overline{\varepsilon }_{p} , T} \right) = \overline{\sigma } - \sigma_{p} \left( {\overline{\varepsilon }_{p} , T} \right) $$

16 $$ \overline{\sigma } = \sqrt {\frac{{\left( {\sigma_{11} - \sigma_{22} } \right)^{2} + \left( {\sigma_{33} - \sigma_{22} } \right)^{2} + \left( {\sigma_{11} - \sigma_{33} } \right)^{2} + 6\left( {\sigma_{12}^{2} + \sigma_{13}^{2} + \sigma_{23}^{2} } \right)}}{2}} $$

where $\overline{\sigma }$ is the equivalent von Mises stress, $\sigma_{p}$ is the HSGPR-based flow stress, $\overline{\varepsilon }_{p}$ is the von Mises equivalent strain, $T$ is temperature, and $\left[ {\sigma_{11} , \sigma_{22} , \sigma_{33} , \sigma_{12} , \sigma_{13} . \sigma_{23} } \right]$ is the Cauchy stress vector.

The yield function with the HSGPR-based flow stress model is called at each increment of the FEA to check the yield state of each element integration point and update the flow stresses of the element integration points which have reached plasticity. In order to update the flow stresses, the stiffness of the HSGPR-based flow stress model, that is the gradient of the flow stress model $$\sigma_{p}$$ with respect to the equivalent plastic strain $$\overline{\varepsilon }_{p}$$ and temperature $$ T{ }$$, respectively, is queried in the FEA^32^. The corresponding stiffness (i.e. $$\partial \sigma_{p} /\partial \overline{\varepsilon }_{p}$$ and $$\partial \sigma_{p} /\partial T$$) of the HSGPR-based flow stress model is expressed as:

17 $$ \frac{{\partial \sigma_{p} }}{{\partial \overline{\varepsilon }_{p} }} = \frac{{\partial \overline{\phi }\left( {\overline{\varepsilon }_{p} ,T } \right)}}{{\partial \overline{\varepsilon }_{p} }}\overline{\user2{w}} + \frac{{\partial \delta \left( {\overline{\varepsilon }_{p} ,T} \right)}}{{\partial \overline{\varepsilon }_{p} }} \cdot {\mathcal{N}}\left( {0,1^{2} } \right) $$

18 $$ \frac{{\partial \sigma_{p} }}{\partial T} = \frac{{\partial \overline{\phi }\left( {\overline{\varepsilon }_{p} ,T } \right)}}{\partial T}\overline{\user2{w}} + \frac{{\partial \delta \left( {\overline{\varepsilon }_{p} ,T} \right)}}{\partial T} \cdot {\mathcal{N}}\left( {0,1^{2} } \right) $$

where the detailed expressions and inferences of the gradients $\partial \overline{\phi }\left( {\overline{\varepsilon }_{p} ,T } \right)/\partial \overline{\varepsilon }_{p}$**,** $\partial \overline{\phi }\left( {\overline{\varepsilon }_{p} ,T } \right)/\partial T$**,** $\partial \delta \left( {\overline{\varepsilon }_{p} ,T} \right)/\partial \overline{\varepsilon }_{p}$ and $\partial \delta \left( {\overline{\varepsilon }_{p} ,T} \right)/\partial T$ are presented in “Appendix B” of the Supplementary Information.

This procedure can be achieved in the general-purpose finite element software Abaqus^33^ via its subroutine UHARD. The numerically implemented HSGPR-based stochastic flow stress model can then be used to solve the temperature-dependent plastic problems.

### Stochastic plastic structural analysis procedure

**Figure 8:**
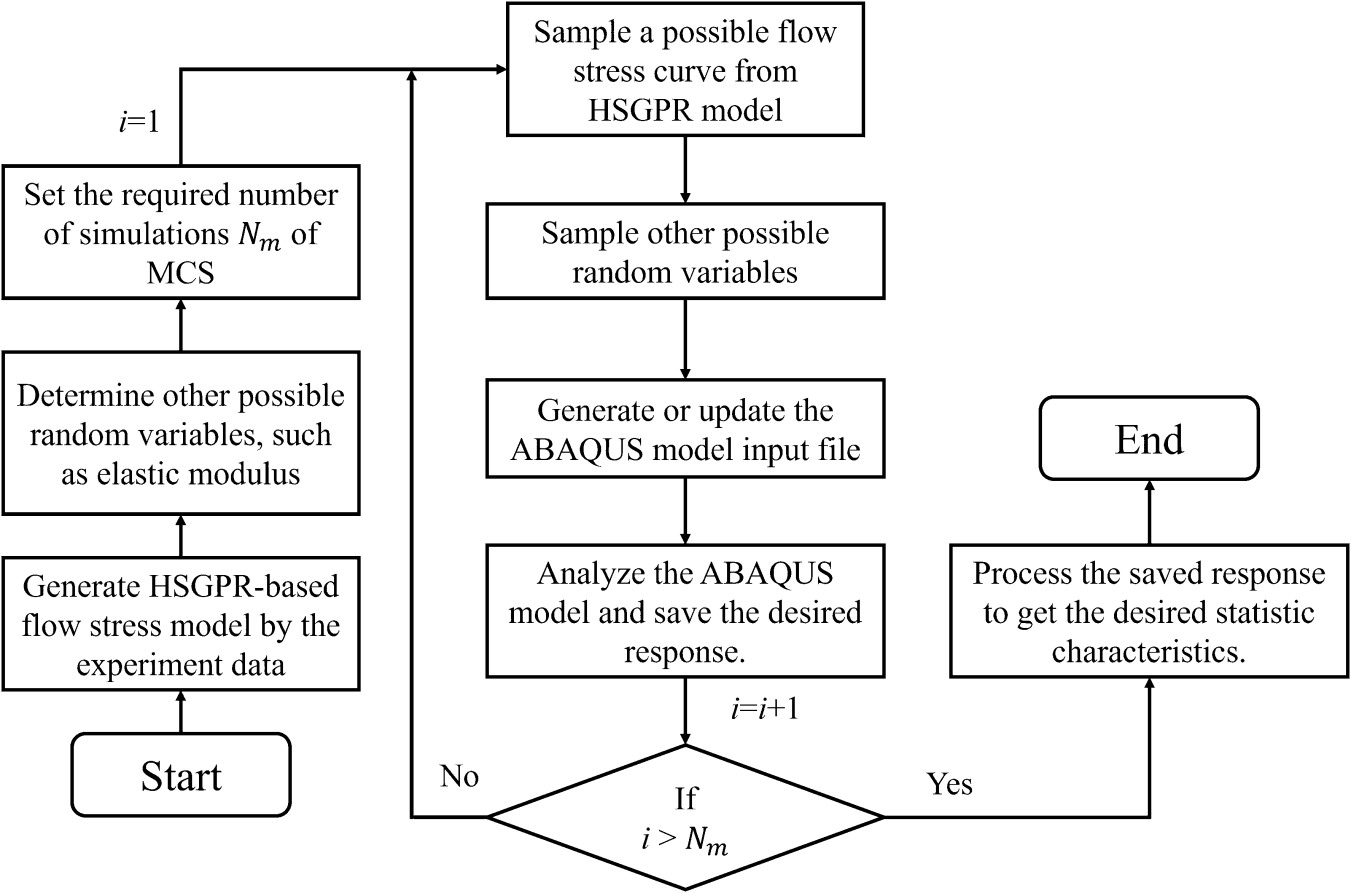
Flowchart of the stochastic plastic structural analysis steps with the HSGPR-based model.

The Monte Carlo Simulation (MCS) method is applied to achieve the stochastic plastic structural analysis using the HSGPR-based model. The possible flow stress relations estimated by the HSGPR model are generated by following the flow chart in Fig. 1 and samples of other possible basic random variables, such as elastic modulus (E), can also be generated according to their probability distribution. For each sample, a finite element model is set up and a complete analysis is performed using Abaqus. The procedure of performing the plastic stochastic structural analysis with the HSGPR-based model is presented schematically in Fig. 8. During each iteration, the sampled flow stress and other possible basic random variables are used to update the corresponding value in the Abaqus input files. The number of the MCS, which can result in a converged estimation, is applied in the procedure for the stochastic structural analysis.

### Numerical examples

#### Beam problem

As a data-driven material model, the HSGPR-based flow stress model should be compatible with both well-known and unknow (or new) constitutive laws. Here, a well-known flow stress model will be used to validate the developed HSGPR-based flow stress model in the stochastic plastic structural analysis. Instead of using the experimental data, the training data for this example are obtained from the Abaqus built-in Johnson Cook model (Eq. 14), which is the target for the HSGPR-based flow stress model to learn. $T_{r}$ and $T_{m}$ of the Johnson Cook model are the deterministic values of 293.15 K and 925 K respectively. The other parameters of the Johnson Cook model are set as the normal random variables to include the effect of the material uncertainty. The means of the parameters $c_{1}$, $c_{2}$, $c_{3}$ and $c_{4}$ of the JC model are 324 MPa, 114 MPa, 0.42 and 1.34, respectively^34^. The coefficients of variation (COV) of four parameters are set as 0.05, 0.10 and 0.15 to consider three different uncertainty levels. Six temperatures (20, 100, 150, 200, 250, 300 °C) are considered in the training dataset. For each temperature, 20 stress–strain curves (24,000 data points), 100 stress–strain curves (120,000 data points), and 200 stress–strain curves (240,000 data points) are generated for studying the effect of the dataset size. Hence, there are totally nine synthetic flow stress datasets with three uncertainty levels and three dataset sizes generated for training the HSGPR model. The Poisson ratio of the material is set as 0.33 and the elastic modulus is set as a temperature-dependent normal random variable^35^ and is expressed as

19 $$ E_{T} = \left( {1 - \frac{{T - T_{r} }}{1600}} \right)E_{0} $$

where $T$ (K) is the temperature, $T_{r}$ is the room temperature 293.15 K and the room temperature elastic modulus $E_{0}$ is a normal random variable with a mean of 69,500 MPa and a standard deviation of 8,548.5 MPa^35^.

**Figure 9:**
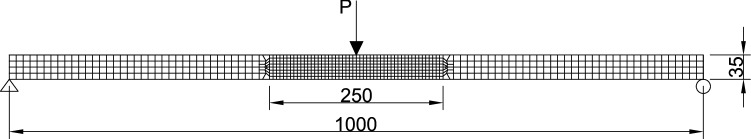
Boundary condition, loading condition, geometrical size (mm), and mesh distribution of the example model.

**Figure 10:**
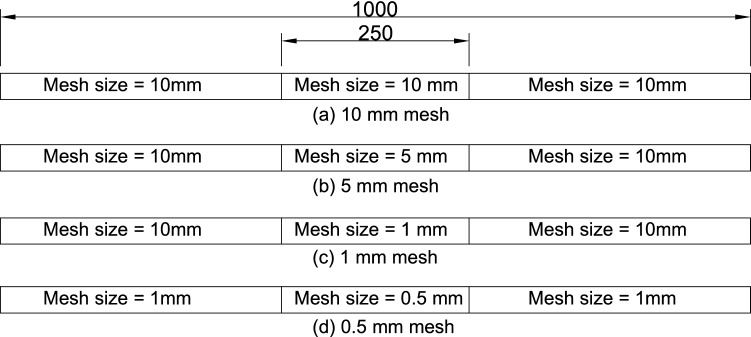
Four different mesh size strategies for the FEA model of the beam. (**a**) 10 mm mesh, (**b**) 5 mm mesh, (**c**) 1 mm mesh, (**d**) 0.5 mm mesh.

**Figure 11:**
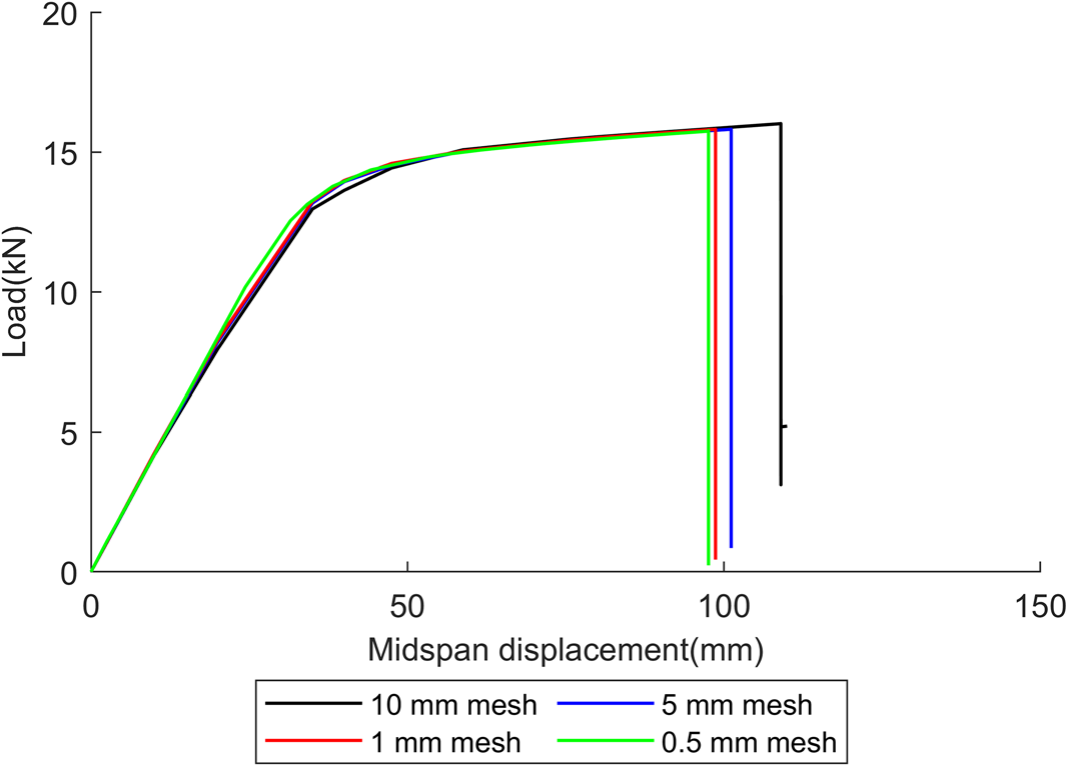
The load–displacement curves of four mesh strategies.

After the training process, the HSGPR-based flow stress model is implemented into the FEA and the model is validated by the beam problem adopted from^36^. The geometry, boundary conditions and loading conditions of the beam are depicted in Fig. 9 and the thickness of the beam is 35 mm. The failure strain is set as 0.1^16^. Except for the elastic modulus and flow stress, other uncertainty sources are not taken into account in the current validation process. Abaqus is used to conduct the finite element analysis. The displacement-controlled loading is applied at the middle point of the beam model. The mesh convergence study is conducted with all the parameters being set to their mean values. The linear 4-node doubly curved shell element (S4R) is used in the Abaqus model. As depicted in Fig. 10, four different mesh size strategies are studied in the present work. The load–displacement curve of each case is presented in Fig. 11. The relative errors of the load capacity (i.e. peak load) of the cases with 10 mm, 5 mm and 1 mm mesh relative to the case with 0.5 mm mesh are 1.62%, 0.35% and 0.32% respectively. The case with 5 mm mesh, which could provide sufficient accuracy for estimating the load capacity and acceptable computational efficiency, is used in the current study. The mesh distribution of the beam is presented in Fig. 9.

**Figure 12:**
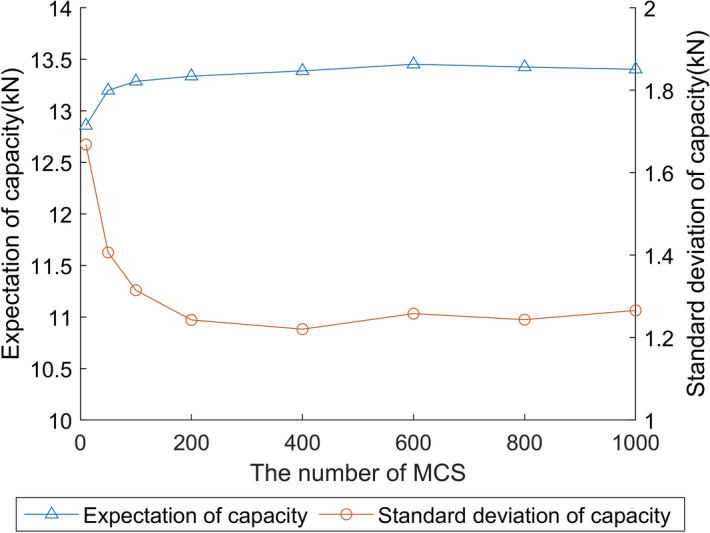
The expectation and standard deviation evaluated by the HSGPR model at the different number of MCS. (Temperature = 175 °C, COV = 0.10, the number of data curves = 100).

In order to determine the number of MCS, the stochastic structural capacity of the beam is evaluated by the HSGPR model at the different number of iterations. The expectation and standard deviation of the structural capacity with respect to the number of MCS is presented in Fig. 12. It can be noted from the figure that both the expectation and standard deviation given by the MCS converge at the number of 1000. Hence, the number of MCS is set as 1000 for the structure analysis.

**Figure 13:**
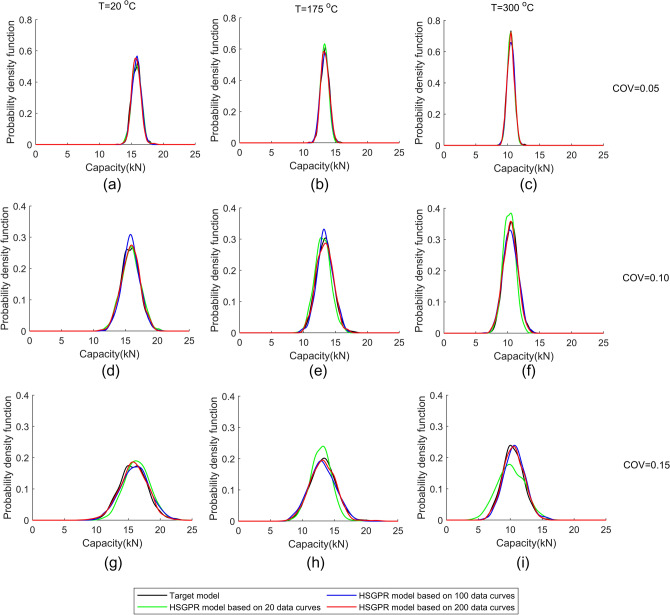
The probability density distribution of the structural capacity given by the HSGPR-based flow stress model and the target model at different dataset size, temperatures and coefficient of variation (COV). (**a**) COV = 0.05, T = 20 °C, (**b**) COV = 0.05, T = 175 °C, (**c**) COV = 0.05, T = 300 °C, (**d**) COV = 0.10, T = 20 °C, (**e**) COV = 0.10, T = 175 °C, (**f**) COV = 0.10, T = 300 °C, (**g**) COV = 0.15, T = 20 °C, (**h**) COV = 0.15, T = 175 °C, (**i**) COV = 0.15, T = 300 °C.

The probability density distributions of the structural load capacities given by the HSGPR-based flow stress model and the targe model at the room temperature and the elevated temperatures are presented in Fig. 13. The corresponding mean and standard deviation of the load capacities are illustrated in Tables 6 and 7, respectively.

**Table 6:** The expectation of the structural capacity given by the HSGPR-based flow stress model and the target model at different dataset sizes, temperatures and coefficient of variation (COV).

COV	Number of data curves	T = 20 °C			T = 175 °C			T = 300 °C		
		Target (kN)	HSGPR (kN)	Relative error (%)	Target (kN)	HSGPR (kN)	Relative error (%)	Target (kN)	HSGPR (kN)	Relative error (%)
0.05	20	15.80	15.82	0.1	13.38	13.30	0.6	10.47	10.47	0.0
	100		15.82	0.1		13.34	0.3		10.49	0.2
	200		15.76	0.3		13.37	0.1		10.47	0.0
0.10	20	15.77	15.84	0.4	13.35	13.02	2.5	10.49	10.24	2.4
	100		15.84	0.4		13.40	0.4		10.43	0.6
	200		15.81	0.3		13.32	0.2		10.40	0.9
0.15	20	15.80	16.36	3.5	13.32	12.91	3.1	10.42	10.11	3.0
	100		16.05	1.6		13.12	1.5		10.68	2.6
	200		15.81	0.1		13.22	0.8		10.52	1.0

**Table 7:** The standard deviation of the structural capacity given by the HSGPR-based flow stress model and the target model at different dataset sizes, temperatures and coefficient of variation (COV).

COV	Number of data curves	T = 20 °C			T = 175 °C			T = 300 °C		
		Target (kN)	HSGPR (kN)	Relative error (%)	Target (kN)	HSGPR (kN)	Relative error	Target (kN)	HSGPR (kN)	Relative error (%)
0.05	20	0.72	0.74	2.8	0.64	0.61	4.7	0.56	0.53	5.4
	100		0.70	2.8		0.66	3.1		0.56	0.0
	200		0.71	1.4		0.64	0.0		0.56	0.0
0.10	20	1.41	1.51	7.1	1.30	1.24	4.6	1.08	0.95	12.0
	100		1.34	5.0		1.27	2.3		1.16	7.4
	200		1.43	1.4		1.27	2.3		1.10	1.9
0.15	20	2.13	1.99	6.6	1.95	1.65	15.4	1.64	2.13	29.9
	100		2.21	3.8		2.03	4.1		1.68	2.4
	200		2.18	2.3		2.00	2.6		1.63	0.6

It can be observed from Table 6 that the expected capacity given by the HSGPR-based flow stress model have high accuracy with the relative error smaller than 4% even under the high uncertainty level and small dataset size. When the dataset size is large enough, the influence of increasing dataset has little effect on the accuracy of expected capacity. The slight error variation may be due to the model itself. For the variability of the load capacity, the probability density distribution obtained by the HSGPR model coincides with the distribution obtained by the target model at the low uncertainty level (COV = 0.05), as shown in Fig. 13. For the high uncertainty level, the HSGPR model requires larger dataset size to achieve good estimation of the COV of load capacity. With the sufficient material data, the relative error of the standard deviation given by the HSGPR model is smaller than 3%. As presented in Tables 6 and 7, the effect of temperature on the accuracy of the HSGPR model is not obvious at all uncertainty levels, which means that the HSGPR-based flow stress model is suitable for the stochastic plastic structural analysis at elevated temperatures with sufficient dataset. In addition, it is worth noting that the HSGPR model can give good estimation on the load capacity at the temperature of 175 °C which is not considered in the synthetic dataset.

#### Punch problem

To evaluate the performance of the HSGPR-based model in the loading and unloading process, the punch problem given by Huang et. al.^37^ is used as the benchmark test. The exponential flow stress law $\sigma_{p} = e_{1} + e_{1} (e_{2} + \varepsilon_{p} )^{{e_{3} }}$ is set as the target model. To consider the material uncertainty, the parameters $e_{1}$, $e_{2}$ and $e_{3}$ are set as the normal random variables with the mean of 0.05 MPa, 0.00002 and 0.3 respectively and the COV of 0.05. A total of 200 stress–strain curves (40,000 data points) at the room temperature are generated for training the HSGPR model. The Poison ratio is set as 0.33 and the elastic modulus is set as the normal random variable with the mean of 1 MPa and the standard deviation of 0.05 MPa.

**Figure 14:**
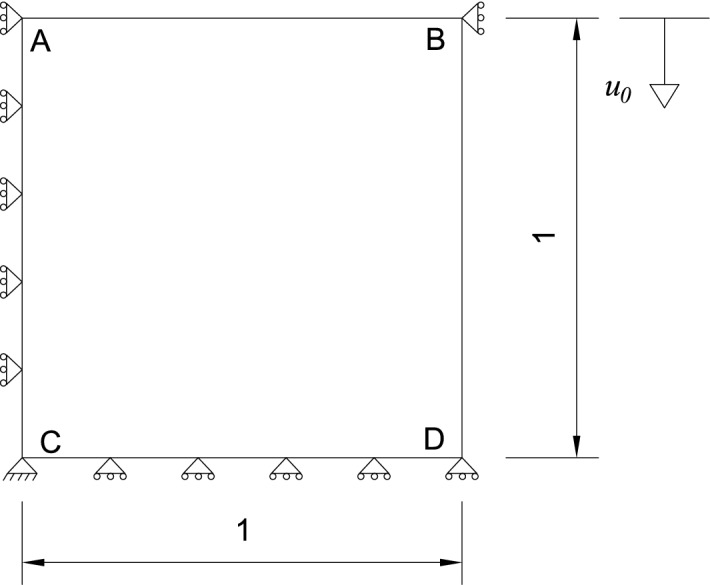
Punch problem given by Huang et. al.^37^ (Unit: mm).

The HSGPR-based flow stress model obtained after the training process is evaluated by the punch problem^37^. The geometry of the problem is depicted in Fig. 14. The left side and the top right corner of the block are only fix in the horizontal direction and the bottom of the block is only fix in the vertical direction. During the loading stage, the vertical displacement of the top of the block u_0_ is gradually increased to 0.07 mm, which can be regarded as a quasi-static process. During the unloading stage, the direction of the displacement is reversed until u_0_ = 0.02 mm. Similar to Huang et. al.^37^, the block is divided into 100 quadratic quadrilateral elements for the FEA analysis. The load–displacement curve of the node A is recorded.

**Figure 15:**
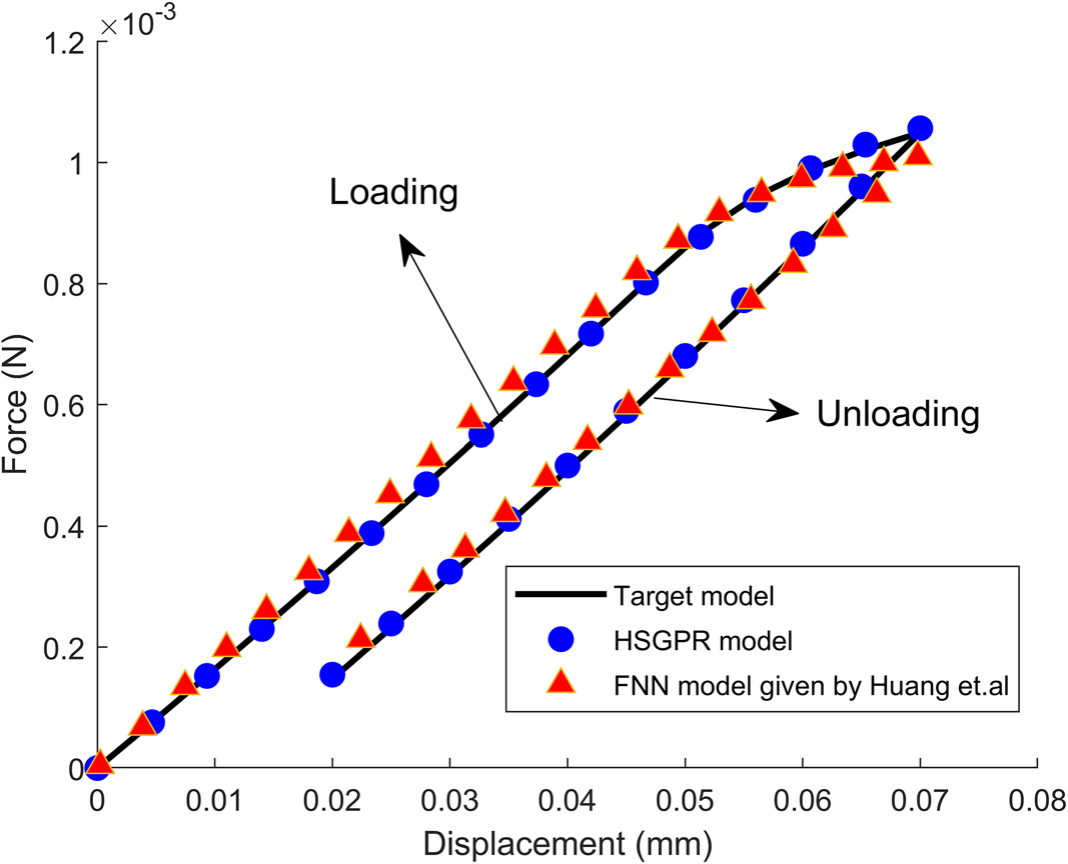
Deterministic load–displacement curves obtained by the target model, HSGPR model and FNN model given by Huang et. al.^37^.

For the deterministic analysis, the obtained load–displacement curve is plotted in Fig. 15. The load displacement simulated by using the feed forward neural network (FNN)-based plasticity model by Huang et al.^37^ is also presented in Fig. 15. It can be observed from Fig. 15 that the HSGPR model and FNN model given by Huang et al.^37^ have the similar performance with both being close to the target model during the loading and unloading processes. And the average relative error of the HSGPR model is 0.75%. However, unlike the HSGPR model, the FNN model cannot capture the material uncertainty and be used in the stochastic plastic structural analysis.

**Figure 16:**
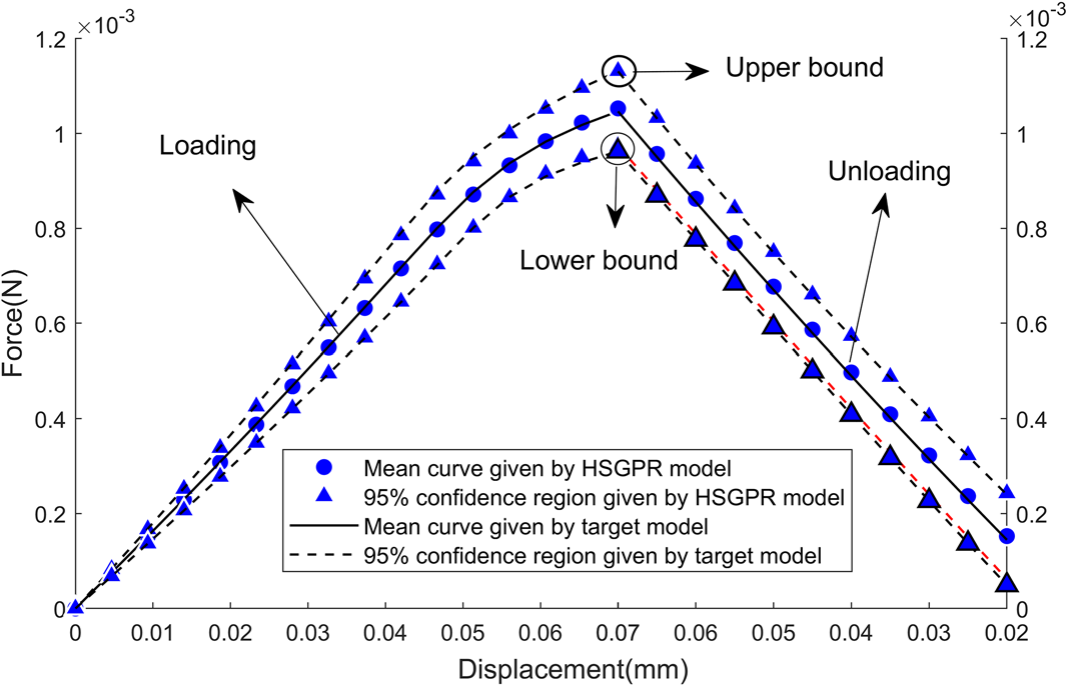
Stochastic load displacement curves obtained by target model, HSGPR model.

Similar to the beam problem, 1000 Monte Carlo simulations are sufficient to provide the converged estimation for the punch problem as well. By conducting 1000 Monte Carlo simulations using the HSGPR model, the 95% confidence region (i.e. ${\text{mean}} \pm 2 \times {\text{standard deviation}}$) of the load–displacement curve can be obtained and is plotted in Fig. 16. As depicted in Fig. 16, the confidence region given by the HSGPR model is in agreement with the target model and the variation of the load–displacement curve is depicted accurately by the HSGPR model in both the loading and unloading stages.

**Figure 17:**
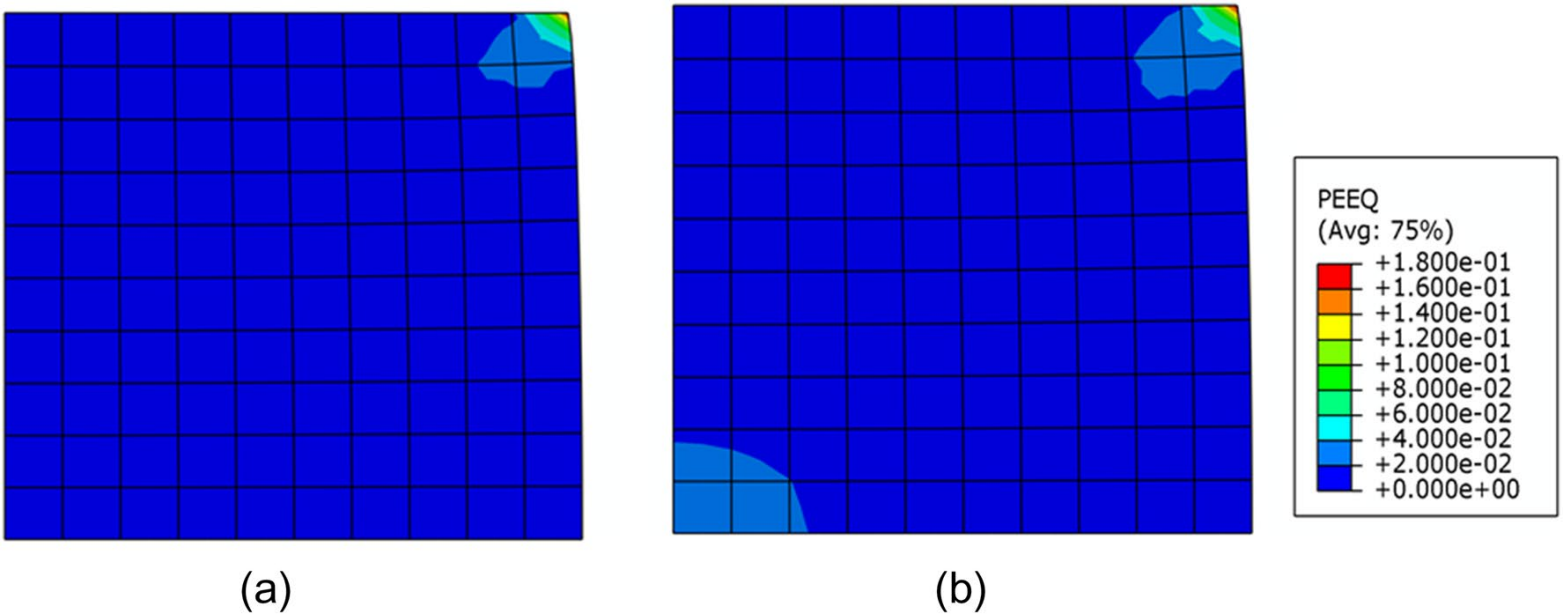
The equivalent plastic strains of the block after loading corresponding to (**a**) the upper bound and (**b**) the lower bound in Fig. 16.

In order to investigate the influence of material uncertainty on the plastic deformation, the equivalent plastic strain of the block after loading is analyzed by using the HSGPR model. Two situations corresponding to the upper bound and the lower bound, as labeled in Fig. 16, are shown in Fig. 17. It can be observed from Fig. 17 that the plastic deformation of the upper bound situation in the lower left conner of the block is smaller than the lower bound situation. However, both the upper bound and the lower bound are the possible situations of the structural responses. The variation of the deformation is caused by the uncertainty inherent in the material. Hence, it can be concluded that the deformation of the structure cannot be described completely without sufficiently considering the flow stress uncertainty.

## Conclusions

Understanding the uncertainty of material flow behavior is important in the stochastic plastic structural analysis. In this paper, a data-driven approach, the heteroscedastic sparse Gaussian process regression (HSGPR)-based flow stress model, is proposed to capture the flow stress behavior and the associated uncertainty directly from the available material data. The established HSGPR-based flow stress model is verified by the experimental data of the Al6061 aluminum alloy and is compared with the conventional GPR model, the ANN model and the Johnson Cook (JC) model. The flow stress of the Al 6061 aluminum alloy at elevated temperatures predicted by the HSGPR model are more accurate than those by the ANN model, the GPR model and the JC model, with the AARE of 6.5% and the MLL of -3.74 on the testing dataset. Besides, unlike the ANN model, which provides only the deterministic flow stress, the flow stress uncertainty is successfully estimated by the HSGPR based model as well.

The HSGPR-based stochastic flow stress model is then implemented into finite element method for stochastic plastic structural analysis. Two numerical examples with synthetic flow stress data, that is the beam problem and the punch problem, are used to verify the accuracy of the HSGPR model in stochastic plastic structural analysis. With the sufficient material data, the expectation and standard deviation of the structural capacity at elevated temperatures have the relative errors less than 1% and 3% respectively in the beam problem. In the punch problem, the variation of the loading and unloading paths given by the HSGPR model is consistent with that given by the target model. The influence of material uncertainty on the structure plastic deformation is successfully identified by the HSGPR model.

## Supplementary Information

Supplementary Information.

## Data Availability

The raw/processed data required to reproduce these findings cannot be shared at this time due to technical or time limitations. However, these data will be shared upon request to the corresponding author.
