# Supplementary material for: Heteroscedastic sparse Gaussian process regression-based stochastic material model for plastic structural analysis
Source: Sci Rep. 2022 Feb 22;12:3017. doi: 10.1038/s41598-022-06870-9 (PMC8864002; doi:10.1038/s41598-022-06870-9)
Supplement: Supplementary file 1 — Supplementary Information. [file 41598_2022_6870_MOESM1_ESM.docx]

**Appendix A**: Optimal psssarameters of the HSGPR model for the Al6061 alloy

$\bar{\boldsymbol{w}}\boldsymbol{=}\left[ \begin{matrix} \begin{matrix} \begin{matrix} 139.8407 \\ 8.4014 \\ -150.6153 \end{matrix} \\ -67.2848 \\ -102.1167 \end{matrix} \\ \begin{matrix} -128.1170 \\ 124.5050 \\ \begin{matrix} 22.2142 \\ 53.4966 \\ -44.6397 \end{matrix} \end{matrix} \end{matrix} \right]\boldsymbol{,}\bar{\boldsymbol{u}}\boldsymbol{=}\left[ \begin{matrix} \begin{matrix} \begin{matrix} 0.4485 \\ 0.0481 \\ 0.0288 \end{matrix} \\ -0.6909 \\ 0.5782 \end{matrix} \\ \begin{matrix} \begin{matrix} -0.7998 \\ -2.0168 \\ 0.4425 \end{matrix} \\ 1.1760 \\ -0.9029 \end{matrix} \end{matrix} \right]$, $\left\{ \boldsymbol{p}_{\boldsymbol{j}} \right\}_{j=1}^{m}\boldsymbol{=}\left[ \begin{matrix} \begin{matrix} \begin{matrix} 0.8661 \\ -1.5756 \\ 0.9295 \end{matrix} \\ -2.0964 \\ \begin{matrix} -0.1183 \\ \begin{matrix} -0.7334 \\ -0.5259 \\ \begin{matrix} 0.2144 \\ 0.3148 \\ 1.8614 \end{matrix} \end{matrix} \end{matrix} \end{matrix} & \begin{matrix} \begin{matrix} -1.4930 \\ -0.5557 \\ 0.9815 \end{matrix} \\ 0.5298 \\ \begin{matrix} -1.5910 \\ \begin{matrix} 2.2147 \\ 2.1055 \\ \begin{matrix} 0.9600 \\ -0.7211 \\ 1.3373 \end{matrix} \end{matrix} \end{matrix} \end{matrix} \end{matrix} \right]$**,** $\left\{ diag(\boldsymbol{\Gamma}_{\boldsymbol{j}}) \right\}_{j=1}^{m}\boldsymbol{=}\left[ \begin{matrix} \begin{matrix} -0.4004 & 0.3178 \\ 1.0143 & -0.5893 \end{matrix} \\ \begin{matrix} -0.3059 & 0.4176 \\ 0.4265 & 0.1901 \end{matrix} \\ \begin{matrix} \begin{matrix} 0.4135 & 0.3017 \\ 0.1211 & 1.3238 \end{matrix} \\ \begin{matrix} -0.2655 & 0.0077 \\ 0.5331 & 0.8298 \end{matrix} \\ \begin{matrix} -0.1921 & 0.6925 \\ 0.6487 & -0.4895 \end{matrix} \end{matrix} \end{matrix} \right]$

$$\bar{b}=6.3003$$

| $\boldsymbol{\Sigma}^{\boldsymbol{-1}}\boldsymbol{=}$ |  | 414.67 | 54.46 | -92.56 | 215.31 | -136.55 | 65.91 | -331.89 | 53.40 | -46.69 | 65.10 |  |
| --- | --- | --- | --- | --- | --- | --- | --- | --- | --- | --- | --- | --- |
|  |  | 54.46 | 8.92 | -8.95 | 23.88 | -17.91 | 8.62 | -41.88 | 6.49 | -7.01 | 5.88 |  |
|  |  | -92.56 | -8.95 | 99.64 | -44.56 | 57.54 | -24.02 | 29.45 | -44.89 | -15.91 | -29.27 |  |
|  |  | 215.31 | 23.88 | -44.56 | 129.66 | -61.80 | 34.22 | -193.08 | 23.83 | -25.89 | 42.10 |  |
|  |  | -136.55 | -17.91 | 57.54 | -61.80 | 64.84 | -23.29 | 79.91 | -29.41 | 4.62 | -22.07 |  |
|  |  | 65.91 | 8.62 | -24.02 | 34.22 | -23.29 | 13.84 | -51.94 | 13.41 | -2.06 | 12.06 |  |
|  |  | -331.89 | -41.88 | 29.45 | -193.08 | 79.91 | -51.94 | 319.85 | -23.66 | 50.13 | -53.22 |  |
|  |  | 53.40 | 6.49 | -44.89 | 23.83 | -29.41 | 13.41 | -23.66 | 21.86 | 6.34 | 13.08 |  |
|  |  | -46.69 | -7.01 | -15.91 | -25.89 | 4.62 | -2.06 | 50.13 | 6.34 | 17.89 | -3.21 |  |
|  |  | 65.10 | 5.88 | -29.27 | 42.10 | -22.07 | 12.06 | -53.22 | 13.08 | -3.21 | 17.83 |  |

$diag(\boldsymbol{\Gamma}_{\boldsymbol{j}})$ denote the horizontal vector storing elements on the diagonal of the matrix $\boldsymbol{\Gamma}_{\boldsymbol{j}}$**.**

**Appendix B**: Inference of the gradients of the HSGPR model

Four terms (${\partial\bar{\boldsymbol{\phi}}\left( \bar{\varepsilon}_{p},T \right)}/{\partial\bar{\varepsilon}_{p}}$**,** ${\partial\bar{\boldsymbol{\phi}}\left( \bar{\varepsilon}_{p},T \right)}/{\partial T}$**,** ${\partial\delta(\bar{\varepsilon}_{p},T)}/{\partial\bar{\varepsilon}_{p}}$ and ${\partial\delta(\bar{\varepsilon}_{p},T)}/{\partial T}$) in Eq. 17 and Eq. 18 need to be inferred for calculating the gradients of the HSGPR-based flow stress model with respected to the equivalent plastic strain $\bar{\varepsilon}_{p}$ and the temperature $T$.

For ${\partial\bar{\boldsymbol{\phi}}\left( \bar{\varepsilon}_{p},T \right)}/{\partial\bar{\varepsilon}_{p}}$**:**

$$\frac{\partial\bar{\boldsymbol{\phi}}\left( \bar{\varepsilon}_{p},T \right)}{\partial\bar{\varepsilon}_{p}}\boldsymbol{=}\left[ \frac{\partial\bar{\phi}_{1}\left( \bar{\varepsilon}_{p},T \right)}{\partial\bar{\varepsilon}_{p}}, \ldots,\frac{\partial\bar{\phi}_{j}\left( \bar{\varepsilon}_{p},T \right)}{\partial\bar{\varepsilon}_{p}},\ldots, \frac{\partial\bar{\phi}_{m}\left( \bar{\varepsilon}_{p},T \right)}{\partial\bar{\varepsilon}_{p}} \right]$$

$$\frac{\partial\bar{\phi}_{j}\left( \bar{\varepsilon}_{p},T \right)}{\partial\bar{\varepsilon}_{p}}\boldsymbol{=}\exp\left[ -\frac{1}{2}\left( [\bar{\varepsilon}_{p},T]-{\bar{\boldsymbol{p}}}_{\boldsymbol{j}} \right)^{T}{\bar{\boldsymbol{\Gamma}}}_{j}^{T}{\bar{\boldsymbol{\Gamma}}}_{j}\left( [\bar{\varepsilon}_{p},T]-{\bar{\boldsymbol{p}}}_{\boldsymbol{j}} \right) \right]\cdot\left[ -{{\bar{\boldsymbol{\Gamma}}}_{j}\left( 1,1 \right)}^{2}\cdot\left[ \bar{\varepsilon}_{p}-\bar{p}_{j}\left( 1 \right) \right] \right]=\bar{\phi}_{j}\left( \bar{\varepsilon}_{p},T \right)\cdot\left[ -{{\bar{\boldsymbol{\Gamma}}}_{j}\left( 1,1 \right)}^{2}\cdot\left[ \bar{\varepsilon}_{p}-\bar{p}_{j}\left( 1 \right) \right] \right]$$

where $\bar{\phi}_{j}\left( \bar{\varepsilon}_{p},T \right)$ is the updated basis function, $\bar{p}_{j}$ and $\bar{\Gamma}_{j}$ are the updated parameters of the basis function $\bar{\phi}_{j}\left( \bar{\varepsilon}_{p},T \right)$.

For ${\partial\bar{\boldsymbol{\phi}}\left( \bar{\varepsilon}_{p},T \right)}/{\partial T}$**:**

$$\frac{\partial\bar{\boldsymbol{\phi}}\left( \bar{\varepsilon}_{p},T \right)}{\partial T}\boldsymbol{=}\left[ \frac{\partial\bar{\phi}_{1}\left( \bar{\varepsilon}_{p},T \right)}{\partial T}, \ldots,\frac{\partial\bar{\phi}_{j}\left( \bar{\varepsilon}_{p},T \right)}{\partial T},\ldots, \frac{\partial\bar{\phi}_{m}\left( \bar{\varepsilon}_{p},T \right)}{\partial T} \right]$$

$$\frac{\partial\bar{\phi}_{j}\left( \bar{\varepsilon}_{p},T \right)}{\partial T}\boldsymbol{=}\exp\left[ -\frac{1}{2}\left( [\bar{\varepsilon}_{p},T]-{\bar{\boldsymbol{p}}}_{\boldsymbol{j}} \right)^{T}{\bar{\boldsymbol{\Gamma}}}_{j}^{T}{\bar{\boldsymbol{\Gamma}}}_{j}\left( [\bar{\varepsilon}_{p},T]-{\bar{\boldsymbol{p}}}_{\boldsymbol{j}} \right) \right]\cdot\left[ -{{\bar{\boldsymbol{\Gamma}}}_{j}\left( 2,2 \right)}^{2}\cdot\left[ \bar{\varepsilon}_{p}-\bar{p}_{j}\left( 2 \right) \right] \right]=\bar{\phi}_{j}\left( \bar{\varepsilon}_{p},T \right)\cdot\left[ -{{\bar{\boldsymbol{\Gamma}}}_{j}\left( 2,2 \right)}^{2}\cdot\left[ \bar{\varepsilon}_{p}-\bar{p}_{j}\left( 2 \right) \right] \right]$$

For ${\partial\delta(\bar{\varepsilon}_{p},T)}/{\partial\bar{\varepsilon}_{p}}$:

$$\frac{\partial\delta\left( \bar{\varepsilon}_{p},T \right)}{\partial\bar{\varepsilon}_{p}}=\frac{\partial\sqrt{v\left( \bar{\varepsilon}_{p},T \right)+\beta\left( \bar{\varepsilon}_{p},T \right)}}{\partial\bar{\varepsilon}_{p}}=\frac{1}{2}\left[ v\left( \bar{\varepsilon}_{p},T \right)+\beta\left( \bar{\varepsilon}_{p},T \right) \right]^{-\frac{1}{2}}\left( \frac{\partial v\left( \bar{\varepsilon}_{p},T \right)}{\partial\bar{\varepsilon}_{p}}+\frac{\partial\beta\left( \bar{\varepsilon}_{p},T \right)}{\partial\bar{\varepsilon}_{p}} \right)$$

$$\frac{\partial v\left( \bar{\varepsilon}_{p},T \right)}{\partial\bar{\varepsilon}_{p}}=\bar{\boldsymbol{\phi}}\left( \bar{\varepsilon}_{p},T \right)\boldsymbol{\cdot}\boldsymbol{\Sigma}^{\boldsymbol{-1}}\boldsymbol{\cdot}\frac{\partial\bar{\boldsymbol{\phi}}\left( \bar{\varepsilon}_{p},T \right)}{\partial\bar{\varepsilon}_{p}}\boldsymbol{+}\frac{\partial\bar{\boldsymbol{\phi}}\left( \bar{\varepsilon}_{p},T \right)}{\partial\bar{\varepsilon}_{p}}\boldsymbol{\cdot}\boldsymbol{\Sigma}^{\boldsymbol{-1}}\boldsymbol{\cdot}\bar{\boldsymbol{\phi}}\left( \bar{\varepsilon}_{p},T \right)$$

$$\frac{\partial\beta\left( \bar{\varepsilon}_{p},T \right)}{\partial\bar{\varepsilon}_{p}}=exp(\bar{\boldsymbol{\phi}}\left( \bar{\varepsilon}_{p},T \right)\bar{\boldsymbol{u}}+\bar{b})\cdot\frac{\partial\bar{\boldsymbol{\phi}}\left( \bar{\varepsilon}_{p},T \right)}{\partial\bar{\varepsilon}_{p}}\boldsymbol{\cdot}\bar{\boldsymbol{u}}$$

For ${\partial\delta(\bar{\varepsilon}_{p},T)}/{\partial T}$:

$$\frac{\partial\delta\left( \bar{\varepsilon}_{p},T \right)}{\partial T}=\frac{\partial\sqrt{v\left( \bar{\varepsilon}_{p},T \right)+\beta\left( \bar{\varepsilon}_{p},T \right)}}{\partial T}=\frac{1}{2}\left[ v\left( \bar{\varepsilon}_{p},T \right)+\beta\left( \bar{\varepsilon}_{p},T \right) \right]^{-\frac{1}{2}}\left( \frac{\partial v\left( \bar{\varepsilon}_{p},T \right)}{\partial T}+\frac{\partial\beta\left( \bar{\varepsilon}_{p},T \right)}{\partial T} \right)$$

$$\frac{\partial v\left( \bar{\varepsilon}_{p},T \right)}{\partial T}=\bar{\boldsymbol{\phi}}\left( \bar{\varepsilon}_{p},T \right)\boldsymbol{\cdot}\boldsymbol{\Sigma}^{\boldsymbol{-1}}\boldsymbol{\cdot}\frac{\partial\bar{\boldsymbol{\phi}}\left( \bar{\varepsilon}_{p},T \right)}{\partial T}\boldsymbol{+}\frac{\partial\bar{\boldsymbol{\phi}}\left( \bar{\varepsilon}_{p},T \right)}{\partial T}\boldsymbol{\cdot}\boldsymbol{\Sigma}^{\boldsymbol{-1}}\boldsymbol{\cdot}\bar{\boldsymbol{\phi}}\left( \bar{\varepsilon}_{p},T \right)$$

$$\frac{\partial\beta\left( \bar{\varepsilon}_{p},T \right)}{\partial T}=exp(\bar{\boldsymbol{\phi}}\left( \bar{\varepsilon}_{p},T \right)\bar{\boldsymbol{u}}+\bar{b})\cdot\frac{\partial\bar{\boldsymbol{\phi}}\left( \bar{\varepsilon}_{p},T \right)}{\partial T}\boldsymbol{\cdot}\bar{\boldsymbol{u}}$$
